# Ulcerative Colitis in Hematological Malignancies: Paraneoplastic Manifestation or Coincidental Bystander?

**DOI:** 10.1155/2020/6135425

**Published:** 2020-03-23

**Authors:** Gregorios Christodoulidis, Konstantinos Perivoliotis, Anastasios Manolakis, Alexandros Diamantis, Apostolos Koffas, Dimitrios Magouliotis, Vasiliki Pappi, Dimitrios Zacharoulis

**Affiliations:** ^1^Department of Surgery, University Hospital of Larissa, Mezourlo 41110, Larissa, Greece; ^2^Department of Gastroenterology, University Hospital of Larissa, Mezourlo 41110, Larissa, Greece; ^3^Department of Hematology, General Hospital of Volos, Volos, Greece

## Abstract

Evidence of coexistence of diverse hematological malignancies—lymphoma, leukemia, multiple myeloma, and myelodysplastic syndromes—and either ulcerative colitis or Crohn's disease can be found in the literature. However, a more “systemic” effort to reach further and examine the potential of either one as paraneoplastic manifestation has not been performed. Based on these, three cases of ulcerative colitis manifesting before, simultaneously, and after the onset of different hematological malignancies are presented and critically evaluated.

## 1. Introduction

The term “paraneoplastic” has been coined to describe a series of manifestations resulting from biologically active substances or immune responses, induced by solid tumors or hematological malignancies. Evidence of coexistence of diverse hematological malignancies—lymphoma, leukemia, multiple myeloma, and myelodysplastic syndromes—and either ulcerative colitis (UC) or Crohn's disease (CD) can be found in the literature [1–3]. However, a more “systemic” effort to reach further and examine the potential of either one as potential paraneoplastic manifestation has not been performed. Based on these, three cases of UC manifesting before, simultaneously, and after the onset of different hematological malignancies are presented and critically evaluated.

## 2. Case Presentation

### 2.1. Case 1

A 71-year-old male Caucasian with a history of multiple myeloma (MM) treated with lenalidomide and dexamethasone was referred to our department with episodes of hematochezia. Lenalidomide was ceased one month ago, due to mild diarrhea. The patient was on a 100 mg/day acetylsalicylic acid thromboprophylaxis regimen. Endoscopic and histopathologic findings indicative of ulcerative pancolitis as well as laboratory parameters are presented in Table 1. A partial response of MM was recorded, and bone marrow biopsy revealed a 15% monoclonal plasmocyte infiltration. The patient was treated with antibiotics, prednisone, and 5-aminosalicylates (5-ASA), reaching clinical remission. Two months later, upper extremity lytic lesions consistent with MM recurrence were detected. When lenalidomide and dexamethasone were reintroduced later, no UC flare-ups were recorded, despite the lack of a more UC-specific therapy, as the patient, on his own initiative, discontinued 5-ASA. During the next four years, both UC and MM remained in remission.

### 2.2. Case 2

A 57-year-old male Caucasian was admitted for fatigue, low-grade fever, and bloody diarrhea. Endoscopic findings and histopathology, consistent with UC pancolitis and laboratory parameters, are included in Table 1. Due to concomitant pancytopenia, a bone marrow biopsy was performed, which established the diagnosis of myelodysplastic syndrome (MDS). More specifically, an intermediate risk I MDS was diagnosed, with a 10% bone marrow infiltration by blast cells with normal karyotype. Following treatment with antibiotics, prednisone and 5-ASA, remission was established also accompanied by an improvement in blood count analyses (Table 1). The patient did not receive any transfusion and was conservatively supported with erythropoiesis-stimulating agents.

### 2.3. Case 3

A 44-year-old male Caucasian was admitted to our department, with low-grade fever, mild diffuse abdominal pain, and bloody diarrhea. Three years ago, due to episodes of mild diarrhea, and after positive bone marrow biopsies, the diagnosis of systemic mastocytosis (SM) was confirmed. In addition to this, UC was diagnosed 2 months prior to current hospitalization. He was under 5-ASA and tapering doses of prednisone. Laboratory, endoscopic, and histopathologic findings are presented in Table 1. The patient exhibited a UC pancolitis for which he received treatment with prednisone, 5-ASA, budesonide enemas, and azathioprine (AZA). After clinical remission, the patient was closely followed being asymptomatic under AZA and 5-ASA. After 12 months, endoscopy for therapeutic evaluation and biopsies were carried out. All findings were once more suggestive of UC, and the possibility of an SM-diseased colon was histopathologicaly ruled out.

## 3. Discussion

A rather serious turmoil has been created with respect to hematological malignancies complicating inflammatory bowel disease (IBD), and although data from large studies indicate an increased risk for UC patients to develop myeloid leukemia, it is not yet clear whether the disease itself or the different therapeutic agents used are to be held accountable [1, 4]. In contrast, little is known about the prevalence of IBD in patients with hematopoietic malignancies [1–3] while the notion that UC may represent a paraneoplastic manifestation in such cases is “terra incognita.” Motivated by the admission in our tertiary center, during a 2½-year period, of three patients with three different hematological malignancies accompanied by UC at an almost identical extent and severity, it seemed fit to further examine this association.

The close link between inflammation and cancer is well studied, with autoimmune disorders predisposing to malignancies and autoimmune phenomena manifesting more frequently on the setting of cancer. This idea has been recently enriched through the recognition of chronic antigenic stimulation as a major player for the onset of diverse malignancies, including hematological as well as autoimmune disorders such as IBD either occurring independently or in conjunction. Immune-stimulating conditions involving activation of immune cells may lead eventually to pro-oncogenic mutations in actively dividing cells while immune dysregulation taking place in the setting of hematopoietic cancer may lead to cross-linking reactions and recognition of endogenous molecules thus triggering several autoimmune phenomena [5–7]. Moreover, the biological pathways involved in hematological malignancies and UC are interlinked, with several mediators of inflammation and cellular populations with an altered response profile being shared among these entities [8]. Indeed, tumor necrosis factor-alpha (TNF-*α*), nuclear factor kappa-light-chain-enhancer of activated B cells (NF-kB), interleukins (IL-1, -5, -6, and -8), angiogenesis factors (i.e., vascular endothelial growth factor (VEGF)), irregular plasmatoid cells inducing pronounced TNF-*α* and reduced interferon-gamma production, and eosinophils are key-components of the pathogenesis of MM, MDS, SM, and UC [3, 7–12]. Further details pertaining to each case are also presented below.

In the first case, UC was associated with MM while its manifestation preceded by almost 2 months the diagnosis of recurrent MM. In addition, adequate “control” of MM was accompanied by a lack of UC relapse. Acting together, the molecular and cellular components described earlier may have favored the onset of UC, before ultimate diagnosis of MM recurrence. At this point, it is important to underline that all cases of IBD accompanying MM pertain to CD alone. In these cases, the treatment regimens did not, however, include lenalidomide, a thalidomide analogue with a×2000 greater potency for TNF*α* inhibition [9]. Thalidomide has been used in some studies for the induction or maintenance of CD remission, whereas no benefits whatsoever were recorded with respect to UC. Based on these, we cannot exclude the possibility that the thalidomide analog may have conditioned the IBD-genic potential of MM, discouraging the onset of CD while leaving intact those biological pathways ultimately leading to the UC phenotype. Likewise, it seemed quite reasonable to include pharmaceutic colitis in differential diagnosis. Against these notions are the observations that during a 4-year follow-up the diagnosis of UC has not changed and, in addition, when lenalidomide was readministered, no adverse events implicating the colon were recorded.

The link of MDS with several autoimmune phenomena is well established. This close association is such that pharmacologically induced suppression of these manifestations is accompanied by partial MDS improvement, as in our case [10]. Another finding replicated in our study is the association of MDS with an extensive UC (pancolitis) [3]. In terms of pathogenesis, impaired mitogenic response, diminished CD4-positive cell numbers, and functional abnormalities in B cell subset accompanied by hyper- or hypogammaglobulinemia or impaired antibody-dependent killing have been recorded in MDS. Similarly, impairment in mitogenic, CD4-positive, and B cell response has been reported in UC patients. Moreover, certain potent inhibitors of hematopoiesis inducing accelerated cell death, such as TNF-*α*, may also be markedly upregulated in both MDS and UC [3, 10]. Hence, it is not surprising that treatment targeting UC and its biological pathways may exert a favorable effect in hematological status of MDS.

As for the cosegregation of SM with ulcerative colitis is not surprising as both disorders may share a common Th2 pathogenetic background. Moreover, the colon may be implicated in mastocytosis, posing a serious challenge in terms of differential diagnosis, as it is mimicking IBD [11]. According to the published literature, the presence of mast cells may modulate IBD through participation in normal immunity, increase in intestinal permeability and sensitivity, eosinophil chemoattraction, release of vasoactive mediators, and superoxide dismutase colonic injury. In this case, the SM-related increased presence of mast cells and their products could have exerted these phenomena, thus disrupting mucosal epithelial barrier, initiating and sustaining an inappropriate inflammatory process subsequently leading to UC [13].

In view of all three cases, patients were male Caucasians with no family history of IBD. A male predominance in MDS, MM, and lately IBD has already been recorded and is probably reflected in our series, too [8, 10, 14]. Likewise, the common ethnic background of all patients most likely results from the Caucasian predominance in our district, although the presence of certain ethnic/race-related genes conferring susceptibility cannot be excluded. An altered immunogenic profile has been documented, as increased levels of immunoglobulins (IgG and IgA) were recorded. Moreover, the notorious autoimmune disorders, including IBD, cross-reactive c-ANCA, a-ANCA, and ASCA were found, suggesting an altered pattern recognition status, targeting both endogenous as well as microbial components [14–16]. What might be of importance is that c-ANCA and ASCA, are not commonly encountered in UC, and their presence has been associated in some cases with distinct UC phenotype—usually pancolitis [15, 16]. Moreover, positivity for these markers has been shown to be a genetic trait, cosegregating in families irrespective of the presence of IBD in individual relatives [14]. Apart from the evident immune dysregulation, the three cases described above seem to share a common immunogenetic background through major histocompatibility complex class I (MHC-I), HLA B^*∗*^ alleles (B07, B35, B51, and B55), known for their involvement in the presentation of intracellular and viral components as well as the attempt of cancer cells to evade the anticancer immune response of T cells, through intensified expression of HLA products, also recorded in patients with hematologic malignancies [17]. The B07, a rather frequent allele, already linked to hematological malignancies [18], is also frequently encountered among Greek UC patients and associated with greater disease extent [19]. The presence of B35 has been documented in cases of cosegregated autoimmune disorders, such as UC and Takayasu arteritis, as well as UC and postinfectious Guillain-Barré syndrome (GBS) [20–23]. B51 shows a strong link with Adamantiades-Behcet [24] while B55 is involved in hepatitis C virus patient progress [25]. Interestingly, all alleles presented above are also classified under the same HLA B7 “supertype” based on their shared peptide binding motifs [26]. What might be of some importance is that all three patients were tested positive for IgG antibodies against EBV, CMV, HSV-1, and VZV. EBV has been associated with hematological malignancies, mainly lymphoma while EBV and CMV were also related to MM [27]. None of the patients, however, had signs of acute infection or viral reactivation and at this point it is impossible to extend this finding as these seropositivities are very common.

When co-evaluating all the data presented above, it is vital to proceed cautiously in order to avoid any misinterpretations. In clinical practice, a paraneoplastic manifestation often serves as an alarm signal for additional work-up aiming at revealing the presence of an underlying malignancy [28]. Based on the available literature, routine investigation for malignant disorders in patients diagnosed with UC, to date, is not justifiable. The association recorded in our cases although thought-provoking is not adequate on its own to change this notion, and it does, however, seem to raise some issues and encourage further hypotheses. For instance, this “paraneoplastic” UC emerged while being tightly linked to diverse malignancies, yet all of a hematologic “origin.” Interestingly, UC manifested in an identical extent and severity in patients bearing HLA B7 supertype components, an identical viral serology profile, while exhibiting an uncommon, for UC, autoimmune serologic pattern (ASCA and c-ANCA in two cases). In addition, this link was further strengthened as a therapeutic benefit was recorded on the malignancy during effective control of UC and inversely on the maintenance of UC remission with successful treatment of the malignancy. Based on the experience drawn from these cases, a clinician should stay alert during differential diagnosis of common conditions in those disorders. Thus, anemia a common manifestation in UC should not be easily attributed solely to the chronicity of the disorder, blood or ferrum loss, malabsorption, and drug-induced or autoimmune hemolysis, especially if accompanied by additional cytopenias. Vice versa, diarrhea in a patient with known hematological malignancy should not be merely attributed to infection or drug toxicity. For the authors, two additional, important, and quite provocative hypotheses remain to be thoroughly examined. Could the association of UC with hematological malignancies be due to a genetic predisposition, implicating the components of the MHC-I, HLA B7 supertype, as in these cases? Similarly, could this “paraneoplastic” extensive UC represent a distinct entity, a unique or specific UC phenotype similar to the one described for GBS associated with *Campylobacter jejuni* infection [20]?

Taking into account the abovementioned evidence, we can safely conclude that the coexistence of inflammatory bowel disease in hematological malignancies patients that present with gastrointestinal tract symptoms should be acknowledged and investigated. Furthermore, in order to enhance the treatment efficacy outcomes and facilitate medical and pathogenetic research, a multidisciplinary and multicenter approach should be applied.

## Figures and Tables

**Table 1:** Laboratory parameters recorded in each patient

	Case 1	Case 2	Case 3
WBC	9500/*μ*L	Pre-t 2580/*μ*L	12000/*μ*L
		Post-t 3500/*μ*L	

Hct	35%	Pre-t 22.9%	36.6%
		Post-t 26.7%	

Plt	WNR	Pre-t 138000/*μ*L	452000/*μ*L
		Post-t 226000/*μ*L	

Reticulocyte count	WNR	Pre-t 0.02 × 10^6^	WNR
		Post-t 0.028 × 10^6^	
ESR (mm)	40	78	38
CRP (mg/dL)	9	13	1.3
IgG (mg/dL)	WNR	WNR	1890
IgA (mg/dL)	WNR	385	363

ASCA (U/mL)	—	62.2 (IgG)	—
	20.9 U/mL (IgA)	38.1 (IgA)	—

ANCA	—	1 : 40 (a-ANCA)	1 : 320 (c-ANCA)
Anti-EBV	+ (IgG)	+ (IgG)	+ (IgG)
Anti-CMV	+ (IgG)	+ (IgG)	+ (IgG)
Anti-VZV	+ (IgG)	+ (IgG)	+ (IgG)
Anti-HSV-1	+ (IgG)	+ (IgG)	+ (IgG)
HLA typing	B35, B55	B51, B55	B07, B35
Cultures (blood/fecal)	Negative	Negative	Negative

Additional	High avidity	Coombs (−)	High avidity
		Anti-platelet antibodies (−)	

Endoscopy	Continuous pattern involvement of all colonic segments' friability, petechial bleeding, edema, and granularity	Continuous pattern involvement of all colonic segments' mucous friability, petechial bleeding, oedema, and granularity	Similar to case 2

Histopathology	Crypt atrophy, crypt abscesses, and LP mononuclear cell infiltration	Similar to case 1	Similar to cases 1, 2

WBC: white blood cell; Hct: hematocrit; Plt: platelet; t: treatment; ESR: erythrocyte sedimentation rate; CRP: C-reactive protein; Ig: immunoglobulin; ASCA: anti-*Saccharomyces cerevisiae* antibodies; ANCA: anti-neutrophil cytoplasm antibodies; EBV: Epstein–Barr; CMV: cytomegalovirus; HSV-1: herpes simplex virus-1; VZV: varicella-zoster virus; HLA: human leukocyte antigen; WNR: within normal range; LP: lamina propria.

## References

[1] Huang L. C., Merchea A. (2017). Dysplasia and cancer in inflammatory bowel disease. *Surgical Clinics of North America*.

[2] Cosnes J. (2017). What should be done in inflammatory bowel disease patients with prior malignancy?. *Digestive Diseases*.

[3] Wang Z., Zhou Y., Liu Y. (2008). Concurrent inflammatory bowel disease and myelodysplastic syndrome: report of nine new cases and a review of the literature. *Digestive Diseases and Sciences*.

[4] Xie J., Itzkowitz S. H. (2008). Cancer in inflammatory bowel disease. *World Journal of Gastroenterology*.

[5] Jin J. J., Butterfield J. H., Weiler C. R. (2015). Hematologic malignancies identified in patients with hypereosinophilia and hypereosinophilic syndromes. *The Journal of Allergy and Clinical Immunology: In Practice*.

[6] Söderberg K. C., Hagmar L., Schwartzbaum J., Feychting M. (2004). Allergic conditions and risk of hematological malignancies in adults: a cohort study. *BMC Public Health*.

[7] Aggarwal B. B., Shishodia S., Sandur S. K., Pandey M. K., Sethi G. (2006). Inflammation and cancer: how hot is the link?. *Biochemical Pharmacology*.

[8] Reynolds G. J., Annis K. A., de Villiers W. J. S. (2007). Review article: multiple myeloma and inflammatory bowel disease. *Digestive Diseases and Sciences*.

[9] Muller G. W., Chen R., Huang S.-Y. (1999). Amino-substituted thalidomide analogs: potent inhibitors of TNF-*α* production. *Bioorganic & Medicinal Chemistry Letters*.

[10] Giannouli S., Voulgarelis M., Zintzaras E., Tzioufas A. G., Moutsopoulos H. M. (2004). Autoimmune phenomena in myelodysplastic syndromes: a 4-yr prospective study. *Rheumatology*.

[11] Bedeir A., Jukic D. M., Wang L., Mullady D. K., Regueiro M., Krasinskas A. M. (2006). Systemic mastocytosis mimicking inflammatory bowel disease: a case report and discussion of gastrointestinal pathology in systemic mastocytosis. *The American Journal of Surgical Pathology*.

[12] He S.-H. (2004). Key role of mast cells and their major secretory products in inflammatory bowel disease. *World Journal of Gastroenterology*.

[13] Boeckxstaens G. (2015). Mast cells and inflammatory bowel disease. *Current Opinion in Pharmacology*.

[14] Neudecker V., Colgan S. P., Eltzschig H. K. (2017). Novel therapeutic concepts for inflammatory bowel disease—from bench to bedside. *Journal of Molecular Medicine*.

[15] Zhou F., Xia B., Wang F. (2010). The prevalence and diagnostic value of perinuclear antineutrophil cytoplasmic antibodies and anti-*Saccharomyces cerevisiae* antibodies in patients with inflammatory bowel disease in mainland China. *Clinica Chimica Acta*.

[16] Xu J., Yang C.-H., Chen X.-Y., Li X.-H., Dai M., Xiao S.-D. (2008). A subset of ulcerative colitis with positive proteinase-3 antineutrophil cytoplasmic antibody. *World Journal of Gastroenterology*.

[17] Bassani-Sternberg M., Barnea E., Beer I., Avivi I., Katz T., Admon A. (2010). Soluble plasma HLA peptidome as a potential source for cancer biomarkers. *Proceedings of the National Academy of Sciences*.

[18] Bae J., Hideshima T., Zhang G. L. (2018). Identification and characterization of HLA-A24-specific XBP1, CD138 (Syndecan-1) and CS1 (SLAMF7) peptides inducing antigens-specific memory cytotoxic T lymphocytes targeting multiple myeloma. *Leukemia*.

[19] Papasteriades C., Spiliadis C., Emmanouilidis A., Papadimitriou C., Economidou J., Manousos O. (1986). Histocompatibility antigens (HLA-A, B) and ulcerative colitis in a Greek population. *Digestion*.

[20] Wachira V. K., Peixoto H. M., de Oliveira M. R. F. (2019). Systematic review of factors associated with the development of Guillain-Barré syndrome 2007–2017: what has changed?. *Tropical Medicine & International Health*.

[21] Mishu B., Blaser M. J. (1993). Role of infection due to *Campylobacter jejuni* in the initiation of guillain-barre syndrome. *Clinical Infectious Diseases*.

[22] Tominaga K., Tsuchiya A., Sato H. (2019). Co-existent ulcerative colitis and Guillain-Barré syndrome: a case report and literature review. *Clinical Journal of Gastroenterology*.

[23] Hokama A., Kinjo F., Arakaki T. (2003). Pulseless hematochezia: takayasu’s arteritis associated with ulcerative colitis. *Internal Medicine*.

[24] Karasneh J., Gül A., Ollier W. E., Silman A. J., Worthington J. (2005). Whole-genome screening for susceptibility genes in multicase families with Behçet’s disease. *Arthritis & Rheumatism*.

[25] Nishiguchi S., Kaneshiro S., Tanaka M. (2003). Association of HLA alleles with response (especially biochemical response) to interferon therapy in Japanese patients with chronic hepatitis C. *Journal of Interferon & Cytokine Research*.

[26] Sette A., Sidney J. (1999). Nine major HLA class I supertypes account for the vast preponderance of HLA-A and -B polymorphism. *Immunogenetics*.

[27] Csire M., Mikala G., Pető M. (2007). Detection of four lymphotropic herpesviruses in Hungarian patients with multiple myeloma and lymphoma. *FEMS Immunology & Medical Microbiology*.

[28] Pelosof L. C., Gerber D. E. (2010). Paraneoplastic syndromes: an approach to diagnosis and treatment. *Mayo Clinic Proceedings*.

